# Evaluating the Anxiolytic Efficacy of Oral Alprazolam in Modulating Vital Parameters During Minor Oral Surgical Procedures: A Quasi-experimental Study

**DOI:** 10.7759/cureus.77651

**Published:** 2025-01-19

**Authors:** Misbah F Mulla, Vaishali Pagare, Govind R Changule, Amol Doiphode, Mazharkhan Mulla, Rohie Jawarker, Deepak Kaul, Seema Gupta

**Affiliations:** 1 Department of Oral and Maxillofacial Surgery, SMBT Dental College and Hospital, Sangamner, IND; 2 Department of Oral and Maxillofacial Surgery, D Y Patil Dental School, Pune, IND; 3 Department of Oral and Maxillofacial Surgery, Maharashtra Institute of Dental Sciences and Research, Latur, IND; 4 Department of Neurosurgery, Grant Government Medical College and Sir J.J. Group of Hospitals, Mumbai, Mumbai, IND; 5 Department of Oral and Maxillofacial Surgery, M. A. Rangoonwala College of Dental Sciences and Research Centre, Pune, Pune, IND; 6 Department of Orthodontics, Kothiwal Dental College and Research Centre, Moradabad, IND

**Keywords:** alprazolam, anxiety, impaction, minor surgical procedures, vital sign

## Abstract

Introduction: Dental anxiety is a prevalent issue among patients undergoing minor oral surgical procedures such as extractions, biopsies, and implant placement. This study aimed to evaluate the impact of oral alprazolam as a premedicative anxiolytic agent on vital parameters, including pulse rate (PR), systolic and diastolic blood pressure (SBP and DBP, respectively), respiratory rate (RR), and oxygen saturation (SpO2), in patients undergoing minor oral surgeries.

Materials and methods: A quasi-experimental study was conducted on patients aged 18-45 years who required minor oral surgical procedures under local anesthesia (LA) and were divided into two groups based on the Dental Anxiety Scale (DAS) scores. Group 1 (anxious; DAS score > 11) received 0.25 mg oral alprazolam 30 minutes pre-operatively, while Group 2 (non-anxious/mildly anxious; DAS score < 10) underwent surgery without alprazolam. Vital parameters were recorded at three intervals: preoperatively (T0), intraoperatively (15 minutes after starting surgery; T1), and postoperatively (30 minutes after surgery; T2). Statistical analyses included independent t-tests and logistic regression analyses to evaluate differences and associations.

Results: A significant reduction in PR, RR, and SBP was observed in Group 1 across all intervals, compared to Group 2, indicating the effectiveness of alprazolam in stabilizing physiological responses. Postoperative SpO₂ levels remained unaffected in both groups, ensuring patient safety. Logistic regression analysis revealed that elevated preoperative PR, SBP, and RR were significant predictors of anxiety, highlighting their role in identifying candidates for anxiolytic premedication.

Conclusion: Premedication with 0.25 mg oral alprazolam effectively alleviated anxiety and stabilized vital parameters during minor oral surgeries, thereby enhancing patient comfort and procedural efficacy. These findings emphasize the importance of incorporating anxiolytic strategies into dental practice to optimize patient care and surgical outcomes. Future studies should explore different doses and broader populations to refine the anxiolytic interventions.

## Introduction

Minor oral surgical interventions, including tooth extractions, biopsies, and implant placement, constitute a fundamental aspect of dental practice and can generate considerable anxiety and stress among patients. Dental anxiety, a prevalent occurrence, can significantly impact the physiological responses of patients, frequently presenting with an elevated heart rate (HR), heightened blood pressure (BP), and irregularities in respiratory patterns. Such alterations in vital signs induced by stress may further complicate the surgical procedure, resulting in discomfort for both the patient and the dental surgeon [1].

Anxiety not only influences the physiological condition of the patient but may also obstruct the provision of effective healthcare services. Elevated levels of anxiety may result in diminished cooperation, increased pain sensitivity, and prolonged recovery. Consequently, the management of anxiety in dental patients is imperative to facilitate seamless surgical procedures. A variety of strategies, including psychological and pharmacological approaches, have been used to mitigate anxiety. Among these strategies, the application of premedication has garnered considerable attention owing to its capacity to pacify patients and stabilize critical physiological parameters [2,3].

Alprazolam, a short-duration benzodiazepine, is frequently utilized as an anxiolytic agent because of its quick onset and comparatively short elimination half-life. By influencing gamma-aminobutyric acid (GABA) receptors within the central nervous system (CNS), alprazolam diminishes neuronal excitability, thus facilitating a tranquilizing effect [4]. Its anxiolytic characteristics make it an appropriate choice for preoperative sedation in individuals undergoing minor surgical procedures. Nevertheless, the influence of alprazolam on essential physiological parameters such as HR, BP, respiratory rate (RR), and oxygen saturation (SpO2) during dental surgery continues to be an area of significant inquiry [5].

Prior research has established the efficacy of benzodiazepines such as alprazolam in mitigating dental anxiety [1,5]. A dosage of 0.75 mg has been identified as effective for cases of moderate to severe anxiety; however, the outcomes associated with doses of 0.50 mg and 0.25 mg yielded inconsistent findings [1]. Additionally, a study conducted by De Witte et al. [6] indicated that a 0.5 mg dose of alprazolam effectively alleviated stress. The objective of this study was to evaluate the influence of oral alprazolam as a premedicative intervention on the vital signs of patients undergoing minor oral surgical procedures. Through this investigation, it was aimed to examine the role of oral alprazolam in reducing stress and anxiety levels in patients undergoing minor oral surgical interventions, thereby enhancing patient compliance.

## Materials and methods

Study design and setting

This quasi-experimental study was conducted as a thesis project on patients who visited the Department of Oral and Maxillofacial Surgery at M.A. Rangoonwala College of Dental Sciences and Research Centre, Pune, India, from March 2019 to January 2020. Ethical committee approval was obtained from the institute before starting the study (MCES/EC/477/2018), which followed the principles of the Declaration of Helsinki. Written informed consent was obtained from all the patients, and confidentiality was maintained.

Eligibility criteria

The patients aged 18-45 years, who were non-allergic to benzodiazepines, required minor oral surgical procedures under local anesthesia (LA), and patients coming under the category of (American Society of Anesthesiologists (ASA) class I) [7] were included in the study. Alcohol users, those with neurological problems, pregnant and lactating females, and those with cardiac or respiratory problems were excluded from this study.

Sample size estimation

G Power software (Heinrich-Heine-Universität Düsseldorf, Düsseldorf, Germany) was used to estimate the sample size. A minimum of 50 samples were estimated considering an effect size of 0.71 with a mean difference of 2.97 beats/min in pulse rate (PR) and a pooled standard deviation of 4.24 beats/min between patients who underwent minor oral surgical treatment with alprazolam and without alprazolam [8]. The power of the study was 80% with an alpha error of 5%.

Methodology

This quasi-experimental study involved the division of patients into two groups based on their anxiety scores using the Dental Anxiety Scale (DAS) questionnaire [9]. The assessment of anxiety levels and neurological examination of the patients was conducted by a neurosurgeon (MM). Patients with a score of more than 11 were classified as anxious, and therefore, were placed in Group 1, where oral alprazolam 0.25 mg was administered 30 minutes before minor oral surgical procedures under LA [5,8]. Patients with scores less than 10 had no to mild anxiety and, therefore, were treated without oral alprazolam (Group 2). Antihistamines, dietary and weight management supplements, and aspirin were prohibited within a 48-hour timeframe. Furthermore, the intake of alcohol and caffeine was limited for a duration of 72 hours, whereas nicotine use was restricted for a period of 30 days. All patients were reported 60 minutes before the surgical procedure.

Vital parameters such as systolic blood pressure (SBP), diastolic blood pressure (DBP), PR, SpO2, and RR were recorded in a semi-supine position while keeping the backrest of the dental chair at a 30° inclination. The vital parameters were noted before starting the procedure (before administration of oral alprazolam in Group 1) (T0), during the surgery (15 minutes after starting the procedure) (T1), and 30 minutes after the surgery (T2). LA was administered with 2% lidocaine and 1:80000 adrenaline solution injected with a disposable plastic syringe (20 mg/mL lidocaine with 0.0125 mg/mL adrenaline hydrochloride) according to the need for surgery and the type of block used. Special postoperative instructions were given to Group 1 patients to avoid driving, cooking, and any such activity that required concentration until 6 hours postoperatively. There were no dropouts, and no adverse events were noted in the study.

Statistical analysis

The collected measurements were systematically entered into a Microsoft Excel spreadsheet version 2010 (Microsoft® Corp., Redmond, WA, USA) and subsequently analyzed using SPSS software (IBM Corp. Released 2013. IBM SPSS Statistics for Windows, Version 23.0. Armonk, NY: IBM Corp.). Descriptive statistics, including frequency distributions, means, and standard deviations were used to summarize the data. The study groups were compared using an independent t-test. The sex-based distribution between the groups was analyzed using the chi-square test. Logistic regression was used to analyze the predictive factor of anxiety. Statistical significance was determined at a threshold of *P *< 0.05.

## Results

The baseline characteristics of the study groups showed no significant differences in sex distribution, with Group 1 comprising 11 (44%) males and 14 (56%) females, and Group 2 comprising 9 (36%) males and 16 (64%) females (*P* = 0.564). Similarly, the mean age was comparable between Group 1 (31.4 ± 7.42 years) and Group 2 (29.96 ± 5.92 years), with no statistically significant difference (*P* = 0.452). However, the mean anxiety score was significantly higher in Group 1 (14.32 ± 2.04) than in Group 2 (4.68 ± 1.11), with a *P* value of 0.001, indicating a notable difference in baseline anxiety levels between the groups. Therefore, the sex or age-based bias was eliminated in the study (Table 1).

**Table 1:** Baseline characteristics of the study groups. a: *P*-value analysed using chi-square test, b: *P*-value analysed using independent Student’s t-test, *P-value<0.05: significant, sex distribution is represented in form of n (%), anxiety score, and age has been represented in the form of mean ± standard deviation (SD).

Parameter	Group 1	Group 2	Statistic	*P*-value
Male N (%)	11 (44)	9 (36)	0.33^a^	0.564
Female N (%)	14 (56)	16 (64)		
Anxiety score (Mean ± SD)	14.32 ± 2.04	4.68 ± 1.11	20.8^b^	0.001*
Age (years) (Mean ± SD)	31.4±7.42	29.96±5.92	0.23^b^	0.452

The comparison of outcome measures between Groups 1 and 2 revealed significant differences across several parameters. Preoperatively, Group 1 exhibited significantly higher values for PR, RR, SBP, DBP, and SpO2 than Group 2 (*P* < 0.05). During treatment, Group 1 showed statistically significant lower PR and RR values than Group 2, while differences in SBP, DBP, and SpO2 were not statistically significant (*P* > 0.05). Postoperatively, there was a significant reduction in SBP in Group 2 compared to Group 1 (*P* = 0.003), while differences in PR, RR, DBP, and SpO2 between the groups were not statistically significant (*P* > 0.05) (Table 2).

**Table 2:** Comparison of outcome measures by independent Student’s t-test between groups. *P-value < 0.05: significant, SpO2: peripheral oxygen saturation; SBP: systolic blood pressure; DBP: diastolic blood pressure; RR: respiratory rate; PR: pulse rate; data is represented in the form of mean ± standard deviation (SD).

Time intervals	Preoperative (T0)				During surgical procedure (T1)				Postoperative (T2)			
Parameters	Group 1	Group 2	t value	*P-*value	Group 1	Group 2	t value	*P*-value	Group 1	Group 2	t value	*P*-value
PR in beats/minute	99.68 ± 9.93	87.6 ± 11.25	4.02	0.001*	82.24 ± 8.78	88.76 ± 11.36	2.27	0.028*	82.8 ± 6.56	80.48 ± 8.79	1.06	0.296
RR in cycles/ minute	20.08 ± 2.23	18.32 ± 2.41	2.68	0.011*	17.64 ± 1.75	19.32 ± 2.91	2.47	0.017*	16.36 ± 2.1	16.96 ± 1.51	1.16	0.252
SBP in mm Hg	137.68 ± 6.77	127.04 ± 6.64	5.61	0.001*	127.52 ± 4.59	130.8 ± 9.56	1.55	0.129	130.48 ± 7.38	123.32 ± 8.47	3.19	0.003*
DBP in mm Hg	89.84 ± 4.08	84.08 ± 7.95	3.22	0.002*	86.08 ± 5.18	85.04 ± 7.14	0.59	0.558	83.92 ± 4.81	82.16 ± 6.9	1.05	0.301
SpO2 in %	98.56 ± 0.58	97.96 ± 1.27	2.14	0.037*	98.56 ± 0.71	98.36 ± 0.7	1	0.332	98.68 ± 0.56	98.64 ± 0.64	0.24	0.814

In Group 1, significant changes were observed in all parameters except SpO2 levels across all time intervals (*P* = 0.001). Post-hoc tests indicated that PR, RR, and SBP decreased significantly from T0 to T1, whereas non-significant changes were noted in DBP and SpO2 (*P* > 0.05). From T1 to T2, there was a statistically significant decrease in RR (*P *< 0.05); however, the remaining parameters showed no significant change (*P* > 0.05). From T0 to T2, all vital parameters showed a statistically significant decrease (*P* < 0.05), except for SpO2 levels.

In Group 2, significant changes were observed in all parameters except DBP across all time intervals (*P* = 0.001). Post-hoc tests indicated that RR and SBP increased significantly from T0 to T1, whereas no significant changes were noted in PR, DBP, and SpO2 levels (*P* > 0.05). From T1 to T2, there was a significant decrease in RR, PR, and SBP (*P* < 0.05); however, DBP and SpO2 levels were not significantly different (*P* > 0.05). From T0 to T2, PR, RR, and SpO2 levels showed statistically significant changes (*P* < 0.05). This finding demonstrated that the administration of oral alprazolam resulted in a statistically significant decrease in anxiety levels, which consequently led to a reduction in RR, PR, SBP, and DBP when comparing the preoperative and postoperative periods. However, it did not have any significant effect on the SpO2 levels (Table 3).

**Table 3:** Comparison of outcome parameters between preoperative (T0), during surgical procedure (T1) and postoperative (T2) within groups. *P-value < 0.05: significant, SpO2: peripheral oxygen saturation; SBP: systolic blood pressure; DBP: diastolic blood pressure; RR: respiratory rate; PR: pulse rate; ANOVA: analysis of variance; data is represented in the form of mean ± standard deviation (SD).

Group	Parameters	ANOVA		Post-hoc Tukey test		
		F value	*P*-value	T0-T1	T0-T2	T1-T2
Group 1	PR in beats/minute	80.43	0.001*	0.001*	0.001*	1.000
	RR in cycles/minute	31.97	0.001*	0.001*	0.001*	0.018*
	SBP in mm Hg	24.38	0.001*	0.001*	0.001*	0.213
	DBP in mm Hg	10.64	0.001*	0.052	0.001*	0.101
	SpO2 in %	0.37	0.696	1.000	1.000	1.000
Group 2	PR in beats/minute	15.73	0.001*	0.059	0.003*	0.001*
	RR in cycles/minute	14.88	0.001*	0.001*	0.017*	0.001*
	SBP in mm Hg	10.88	0.001*	0.035*	0.188	0.001*
	DBP in mm Hg	1.51	0.231	1.000	1.000	0.152
	SpO2 in %	5.10	0.010*	0.288	0.03*	0.269

Logistic regression analysis to predict high anxiety levels revealed significant associations with certain preoperative parameters. PR demonstrated a significant positive association (*P* = 0.017) with an odds ratio of 1.16, indicating a higher likelihood of high anxiety levels with increasing PR. SBP also showed a significant positive relationship (*P* = 0.002) with an odds ratio of 1.54. While RR approached significance (*P* = 0.089), DBP was not significantly associated with RR (*P* = 0.459). This showed that if the patient had increased PR, SBP, and RR preoperatively, the person was experiencing high anxiety levels, and was an ideal candidate for premedication with 0.25 mg oral alprazolam 30 minutes before the procedure (Table 4).

**Table 4:** Logistic regression for anxiety level (high level used as predicted). *P-value < 0.05: significant, SpO2: peripheral oxygen saturation; SBP: systolic blood pressure; DBP: diastolic blood pressure; RR: respiratory rate; PR: pulse rate; LL: lower limit; UL: upper limit

Variables	Coefficient B	z value	*P-*value	Odds ratio	95% confidence interval (LL-UL)
Constant	-72.27	3.52	0.001	0	0-0
RR in per minute at T0	0.46	1.7	0.089	1.59	0.93-2.72
PR in beats/minute at T0	0.15	2.38	0.017	1.16	1.03-1.31
SBP in mm Hg at T0	0.43	3.14	0.002	1.54	1.17-2.01
DBP in mm Hg at T0	-0.08	0.74	0.459	0.92	0.74-1.14

## Discussion

The current study examined the impact of oral alprazolam as a premedicative intervention on essential physiological parameters in individuals undergoing minor oral surgical interventions. The results of our study revealed that premedication with 0.25 mg of oral alprazolam markedly decreased anxiety levels, as evidenced by the reduction in vital parameters including SBP, DBP, PR, and RR, while maintaining stable SpO2 levels. These findings underscore the clinical relevance of alprazolam for improving patient comfort and promoting more efficient surgical procedures. Our findings are in agreement with those of previous studies [8,10].

Dental anxiety is an extensively examined phenomenon that profoundly affects the physiological state of individuals receiving dental care. Elevated anxiety levels led to a surge in autonomic nervous system activity, resulting in heightened PR, BP, and RR, as demonstrated in the baseline evaluations of Group 1 patients in this study. These findings are consistent with prior empirical research emphasizing the physiological consequences of anxiety experienced during dental procedures [1,5]. Muglali and Komerik conducted a study to assess the factors that increase the anxiety levels of patients preoperatively and concluded that the complexity of the procedure and previous experience were the most common factors [11]. Elevated levels of anxiety not only undermine patient comfort, but also hinder the surgical procedure, thereby potentially elevating the likelihood of unfavorable outcomes and prolonging recovery. This highlights the critical need for effective identification and management of dental anxiety [2].

Alprazolam, a short-duration benzodiazepine, exerts its anxiolytic properties through the modulation of GABA receptors within the CNS. The current investigation substantiates the anxiolytic effectiveness of alprazolam, as demonstrated by a statistically significant decrease in anxiety levels and critical parameters in Group 1 compared to Group 2. Notably, PR, SBP, DBP, and RR exhibited marked reductions from the preoperative to postoperative phase in patients administered alprazolam. These results are consistent with prior research that has emphasized the significance of benzodiazepines in alleviating anxiety during dental and medical interventions [1,6].

The findings of this investigation further illustrated that in Group 1, there was a notable decline in PR, RR, and SBP from the preoperative phase to the postoperative phase. The observed decrease in PR and RR during the surgical procedure (T1) and in the postoperative period (T2) signifies a tranquilizing effect of alprazolam, likely due to its properties as a CNS depressant. This observation holds clinical significance because it indicates that alprazolam has the potential to stabilize the physiological condition of the patient, thereby reducing procedural anxiety and improving surgical efficacy [12]. Additionally, SpO2 levels remained stable across all time intervals in both groups, indicating that alprazolam did not adversely affect SpO2. This is a critical consideration in ensuring patient safety during sedation.

In Group 2, significant increases in RR and SBP were observed during the surgical phase (T1), followed by a reduction in these parameters after surgery (T2). These trends reflect the physiological stress response to surgery, in the absence of anxiolytic premedication. Anxiety experienced during oral surgical procedures can adversely affect patient homeostasis, complicate the execution of the procedure, and induce supplementary stress in the operating surgeon. Moreover, it has been correlated with heightened and enduring pain experienced both during and subsequent to dental interventions [13,14].

Postoperatively, Group 2 showed a significant reduction in SBP compared to Group 1. This finding could be attributed to the natural resolution of surgical stress in the absence of pharmacological interventions. Logistic regression analysis revealed significant associations between the preoperative parameters (PR, SBP, and RR) and high anxiety levels. This finding is clinically significant, as it provides a simple, objective method for identifying patients who may benefit from pharmacological anxiety management. Hollander et al. [15] reported a significant positive correlation between HR and anxiety levels in patients. Astudillo et al. [16] found a positive correlation between anxiety levels and BP, mainly DBP. However, in our study, a positive correlation between SBP and anxiety level was observed. This could have been due to the fact that Astudillo et al. [16] conducted their study in adolescents of age less than 18 years.

Clinical implications of the study

The results of this study have several significant clinical implications. Firstly, in the context of patient selection, identifying individuals exhibiting elevated preoperative PR, SBP, and RR can assist healthcare professionals in administering anxiolytic interventions with greater precision. Secondly, the administration of alprazolam as a premedication alleviates anxiety and stabilizes vital signs, thereby enhancing patient comfort and adherence during minor oral surgical interventions. This enhancement can result in a more favorable overall experience for both patients and healthcare providers. Thirdly, the physiological stability conferred by alprazolam reduces the likelihood of intraoperative complications, enabling dental surgeons to execute procedures with improved efficacy and assurance. The application of 0.25 mg alprazolam in this study offers a reasonable dosage for the management of moderate-to-severe anxiety.

Limitations of the study

The quasi-experimental design and convenience sampling method may have introduced selection bias, limiting the generalizability of the findings. Additionally, the study was conducted in a single institution, which may restrict its applicability to a broader population. Future research could address these limitations by employing randomized controlled trials with larger and more diverse samples. Furthermore, comparative studies evaluating different doses of alprazolam could help to identify the most effective dose for managing dental anxiety. The lack of direct psychological assessment tools, such as the visual analogue scale (VAS-A) or numeric rating scale (NRS), limits the ability to evaluate subjective anxiety changes.

## Conclusions

In conclusion, this investigation illustrates that premedication with 0.25 mg oral alprazolam markedly alleviated anxiety levels and stabilized vital signs in individuals undergoing minor oral surgical interventions. PR, SBP, and RR emerged as significant indicators for evaluating anxiety levels preoperatively. These results underscore the need to incorporate anxiolytic strategies into dental practice to address the challenges associated with dental anxiety. Subsequent research should aim to refine and broaden these approaches to further enhance patient care in the field of oral surgery.
